# Potentiality of Native Ascomycete Strains in Bioremediation of Highly Polychlorinated Biphenyl Contaminated Soils

**DOI:** 10.3390/microorganisms9030612

**Published:** 2021-03-16

**Authors:** Joaquim Germain, Muriel Raveton, Marie-Noëlle Binet, Bello Mouhamadou

**Affiliations:** Laboratoire d’Ecologie Alpine, UMR 5553 CNRS/USMB Université Grenoble Alpes, CEDEX 9, 38058 Grenoble, France; joaquim.germain@univ-grenoble-alpes.fr (J.G.); muriel.raveton@univ-grenoble-alpes.fr (M.R.); marie-noelle.binet@univ-grenoble-alpes.fr (M.-N.B.)

**Keywords:** mycoremediation, native Ascomycetes, PCBs, bioaugmentation, soil functioning

## Abstract

Polychlorinated biphenyls (PCBs) are organic pollutants that are harmful to environment and toxic to humans. Numerous studies, based on basidiomycete strains, have reported unsatisfactory results in the mycoremediation of PCB-contaminated soils mainly due to the non-telluric origin of these strains. The abilities of a five-Ascomycete-strain consortium in the mycoremediation of PCB-polluted soils and its performance to restore their sound functioning were investigated using mesocosm experiments associated with chromatography gas analysis and enzymatic activity assays. With the soil H containing 850 ppm PCB from which the strains had been isolated, a significant PCB depletion of 29% after three months of treatment was obtained. This led to an important decrease of PCBs from 850 to 604 ppm. With the soil L containing 36 ppm PCB, biodegradation did not occur. In both soils, the fungal biomass quantified by the ergosterol assay, did not increase at the end of the treatment. Biodegradation evidenced in the soil H resulted in a significantly improved stoichiometry of N and P acquiring enzymatic activities. This unprecedented study demonstrates that the native Ascomycetes display remarkable properties for remediation and restoration of functioning of the soil they originated from paving the way for greater consideration of these strains in mycoremediation.

## 1. Introduction

Polychlorinated biphenyls are organochlorine compounds consisting of a biphenyl core substituted by one to 10 chlorine atoms with 209 possible congeners differing in the number of chlorine atoms and their position on the biphenyl core [1]. These synthetic compounds have been largely used in industrial products due to their thermal and chemical stability, dielectric properties and low flammability [1,2]. PCBs are among the most persistent pollutant groups widespread in all ecosystems [3,4,5,6,7,8]. They have become a global problem due to their high adsorption capacity in soil and sediment, to their low water solubility, to their capacity to bioaccumulate in fatty tissues, and to their toxicity to animals and humans [9,10,11,12].

Thus, the removal of PCBs from polluted soils represents a major ecological challenge. Since physicochemical methods such as incineration or thermal desorption are expensive and energy-consuming [13], the development of alternative low-cost and environmentally sound strategies also able to restore the soil functionality is needed. To be relevant, such strategies have to allow significant reduction of soil PCBs while ensuring a better nutritional and metabolic equilibria of soil microbial communities. The use of fungal species could be a promising approach. Basidiomycete ligninolytic fungi, naturally associated with wood decay, are the most explored fungi in PCB remediation [2,14,15]. Despite their effectiveness in the PCB degradation in liquid medium, their PCB biodegradation rates in soils which are environmentally different from their natural habitats, were relatively low and seemed to be dependent on the soil PCB concentrations. For instance, Stella et al. (2017) [4] evidenced that the biodegradation rates in soils contaminated with Delor 103 (mix of PCBs containing at least 59 congeners) obtained with *Pleurotus ostreatus* varied and depended on their PCB contents, 18.5%, 41.3%, and 50.5% using soils contaminated at 706 ppm, 376 ppm, and 169 ppm, respectively. These limits are probably due to the weak ability of ligninolytic fungi to develop on PCB-polluted soils probably resulting from their lack of competition with indigenous microbiota [16].

The exploration of the biodegradation abilities of Ascomycete strains isolated from PCB-polluted soils could prove to be relevant. Ascomycetes are the most important fungal group in environment accounting for about 64% of all described fungi [16]. They have been described as able to degrade various environmental pollutants [16,17,18,19,20]. Ascomycete strains isolated from PCB polluted soils are naturally selected by their habitat and potentially able to feed on these pollutants. Similarly to ligninolytic strains, they are able to degrade PCBs efficiently in a liquid medium probably through constitutive enzymes or PCBs-inducible enzymes [21,22]. Despite these interesting potentials, only one study has appraised the biodegradation of PCBs in soils and sediments by native Ascomycete strains [5]. This study, showing that the consortium of six strains was able to degrade PCBs at rates of 18% and 33% when using soils and sediments with total PCB concentration of 400 ppm (15 congeners) and 3.5 ppm (15 congeners), respectively, suggests that these fungi may be prime candidates in the field of environmental biotechnology.

The aim of this study was to investigate the bioremediation potential of five native Ascomycete strains in mesocosm experiments. Two long-term polluted soils with different PCB concentrations were used and one of these soils was that from which the fungal strains had previously been isolated [17]. The abilities of these strains to degrade PCBs, to colonize efficiently both soils as well as to restore their functioning were investigated. 

## 2. Materials and Methods

### 2.1. PCB Congeners Analysed

All analyses focused on the 7 indicator PCBs: PCB 28 (2,4,4′ Trichlorobiphenyl), PCB 52 (2,2′,5,5′-Tetrachlorobiphenyl), PCB 101 (2,2′,4,5,5′-Pentachlorobiphenyl), PCB 118 (2′,3,4,4′,5′-Pentachlorobiphenyl), PCB 138 (2,2′,3,4,4′,5′-Hexachlorobiphenyl), 153 (2,2′,4,4′,5,5′-Hexachlorobiphenyl), and 180 (2,2′,3,4,4′,5,5′-Heptachlorobiphenyl).

### 2.2. Soil Sampling

The studies were carried out on two different PCB-polluted soils: the soil from which the 5 fungal strains had previously been isolated (soil H; Σ7 indicator PCBs: 850 ± 89.0 ppm) [17] and the soil containing a lower PCB-concentration (soil L; Σ7 indicator PCBs: 36 ± 1.8 ppm). Both soils were excavated soils (excavation depth of approx. 50 cm) from two old storage site for electrical materials in France where an electrical transformer containing PCBs had been vandalized. The soil characteristics are shown in Table 1. Each soil sample was sieved (<4 mm) and homogenized by mixing several times prior the remediation treatments.

### 2.3. Inoculum Preparation

The five fungal strains namely *Penicillium chrysogenum, Penicillium canescens*, *Penicillium citreosulfuratum*, *Aspergillus jensenii*, and *Acremonium sclerotigenum* previously isolated [17] are preserved in the laboratory collection (CMPG, Collection Mycology Pharmacy Grenoble). The mycelium (1 g) of each strain, grown for seven days in Petri dishes containing potato dextrose agar and scraped with a scalpel, was ground 10 s with an Ultra Turrax homogenizer (IKA T8, Staufen, Germany) in 50 mL of sterile modified Galzy and Stominsky medium [23]. After checking the cell integrity through microscopy observations, fungal suspensions of the five strains were added to the same sterile chopped straw (30 g) in an autoclave bag to maintain sterility and humidity until colonization of the entire straw. After 14 days at 26 °C, the colonized straw was used for mesocosm experiments.

### 2.4. Design of Mesocosm Experiments

For each soil, biotreatment was performed in mesocosms consisting of glass recipients. Homogenized soils were amended with two preselected surfactants which are soya lecithin (Louis François, 1 g kg^−1^ of dry soil mix) [24] and rhamnolipids (Merck, 0.4 g kg^−1^ of dry soil mix) [25]. Each mesocosm contained 400 g of amended polluted soil which were mixed with 48–50 g of loam (Gamm vert, France) (to reach 10% of organic matter) and 8 g of colonized straw (bioaugmented mesocosms) or non-colonized straw (control mesocosms). The mesocosms were incubated at 25 °C for three months (under natural lighting, ambient humidity), stirred once a week, watered to maintain the studied soils at constant 30% moisture contents. All the experiments were performed in triplicate giving a total of 12 mesocosms (3 replicates × 2 treatment conditions × 2 soil types).

### 2.5. PCB Extraction and Determination of PCB Concentration in Soils

PCBs were extracted from 100 g of soils using hexane/acetone (50/50; *v*/*v*) and analysed by gas chromatography-mass spectrometry by Eurofins Scientific (Saverne, France) according to the standard NF EN 16,167 [26]. 

### 2.6. Fungal Concentrations in Soil

The protocol concerning the soil fungal ergosterol extraction, previously described by Gong et al. (2001) [27] has slightly been modified. Succinctly, 4 g of dry soil from each mesocosm were suspended in 6 mL of methanol and vortexed for 10 s. A soil suspension in methanol was shaken for 1 h at 320 rpm on an orbital shaker then decanted for 15 min. 1.5 mL of supernatant was recovered, placed in a 3 mL Eppendorf microtube and centrifuged for 10 min at 11,000× *g* rpm and 5 °C. A supernatant of 0.9 mL was then filtered through 0.2 µm filters. Finally, a fraction of 0.5 mL of filtrate was evaporated at room temperature and resuspended in 500 µL of dichloromethane.

Ergosterol quantification was performed in the “Institut de Chimie Moléculaire de Grenoble”. Aliquots of 1 µL from each extract were analyzed using a gas chromatograph (5977–7890 B), equipped with a HP-5MS 5 phenyl methyl silox column (ID: 0.25 mm, Length: 30 m, Film: 0.25 mm) and a quadrupole detector (Agilent Technologies, Santa Clara, CA, USA). The carrier gas was helium. The injector temperature and the transfer line temperature were 250 °C and 280 °C, respectively. The initial column temperature was 100 °C held for 3 min. The gradient setting was 50 °C/min to 300 °C, held for 8 min at 300 °C. The applied method of ergosterol analysis was validated on the basis of an internal standard, pregnenolone Acetate (Sigma Aldrich Corp., St. Louis, MI, USA).

### 2.7. Microbial Extracellular Catabolic Enzymatic Activities

Standard fluorimetric methods were used to measure potential extracellular activity of enzymes degrading C-rich substrates (BG = β-1,4-Glucosidase), N-rich substrates: (NAG = β-1,4-N-acetylglucosaminidase; LAP = leucine aminopeptidase) and P-rich substrates: (PHOS = phosphomonoesterase) [28]. Succinctly, 2.75 g of thawed soil were homogenized (1 min in a Waring blender) in 200 mL of a sodium acetate buffer solution adjusted to soil pH (8.1 ± 0.2). The soil slurry (800 µL) was then transferred to a 96-deep-well microplate and added with 200 µL of substrate specific to the four target enzymes at saturation concentration. For each soil sample, duplicated standard curves (0–100 µM concentration) in 96-deep-well microplates were performed by preparing a mixture made of 800 µL of soil slurry and 200 µL of 4-Methylumbelliferone (MUB) on the one hand or 800 µL of soil slurry and 200 µL of 7-amino-4-methylcoumarin (MUC) on the other hand. After incubating at 20 °C in the dark (3 h) on a rotary shaker (150 rpm), plates were subjected to centrifugation (2900× *g*; 3 min). The supernatant (250 µL) was then introduced into a black Greiner flat-bottomed plate and fluorescence was measured on a Varioskan Flash (Thermo Scientific) with excitation wavelength set to 365 nm and emission set to 450 nm. After blank subtraction, potential enzymatic activities were measured out and expressed as nmol g^−1^ soil h^−1^.

### 2.8. Statistical Analyses

Normality of the data was tested by Shapiro-Wilk test. Accordingly, the degradation of total PCBs, the soil ergosterol and the ratios of enzymatic activities were analyzed by Student test while the degradation of each PCB congener was analyzed by Welch test. The analyses of the relationships between soil type and PCB congener concentrations were assessed by two-way ANOVA. Significance levels are always expressed as a value of *p* < 0.05. All statistical analyses were performed using R package version 4.0.4 [29].

## 3. Results

### 3.1. PCB Degradation in Soil

**Figure 1:**
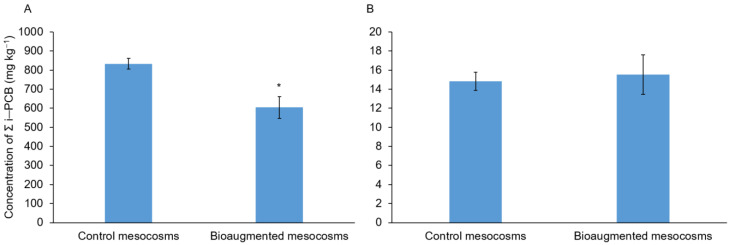
Concentrations of the seven PCBs in mesocosms after a three-month-treatment. (**A**) mesocosms with the soil H and (**B**) mesocosms with the soil L. Bars indicate mean ± standard deviation (*n* = 3). Asterisk indicates significant differences between treatment (Student test; *p*-value < 0.05).

**Figure 2:**
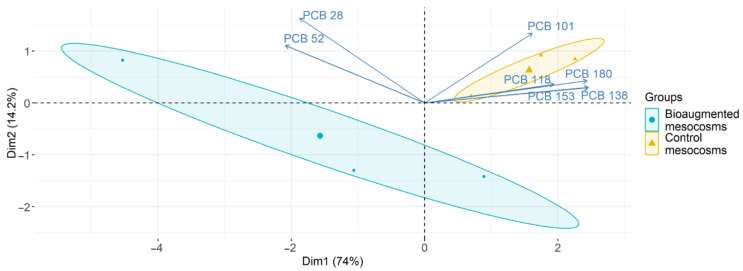
Principal component analysis (PCA) of PCB concentrations in control and bioaugmented soil after a three-month-treatment. The analyses are based on the concentration of seven PCB congeners in control mesocosms (triangle) and bioaugmented mesocosms (circles).

The five fungal strains previously selected for their biodegradation efficiency in liquid medium were used in mesocosm experiments with two different soils. Concerning the soil H, a significant depletion of 29% (*p*-value = 0.04233) of the seven PCBs was observed in the bioaugmented mesocosms after a three-month incubation period compared to the control mesocosms (Figure 1). This depletion was mainly due to a significant decrease (39%) of the PCB 101 concentration (Table 2). The concentration of the six other congeners did not significantly vary between both mesocosms, even if a trend towards a reduction of PCBs 118, 138, 153, and 180 and an increase of the less chlorinated PCBs (PCBs 28 and 52) in the bioaugmented mesocosms was observed (Table 2). PCA analysis (Figure 2) based on the concentrations of PCB congeners in bioaugmented mesocosms and in control mesocosms confirmed this trend. Vertical axis (Dim1, 74%) partially separated the bioaugmented mesocosms from control mesocosms while the horizontal axis (Dim2, 14.2%) separated PCB congeners according to their tendency to increase or decrease in bioaugmented mesocosms. This result was further confirmed by statistical analysis which showed that the relationships between treatments (soil type) and congener concentrations were significant (*p*-value = 0.0235) (Table S1).

Concerning the soil L, there was no significant variation in PCB concentrations between bioaugmented and control mesocosms after a three-month incubation period (Figure 1B, Table 3) suggesting the lack of PCB biodegradation in the bioaugmented mesocosms.

### 3.2. Soil Fungal Colonization

**Figure 3:**
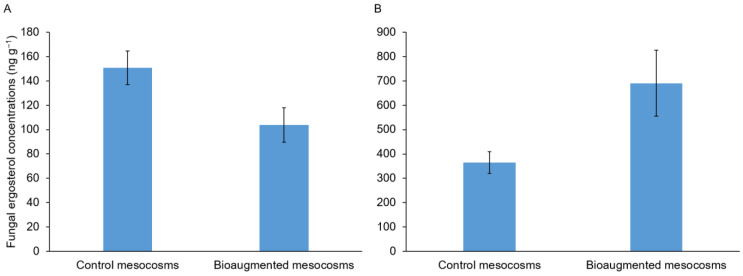
Fungal ergosterol concentrations (ng g^−1^ soil) in the soil H (**A**) and in the soil L (**B**). Bars indicate mean ± standard deviation (*n* = 3); (Student test; *p*-value = 0.1230 (**A**) and 0.1349 (**B**)).

The contribution of bioaugmentation of the total fungal biomass in the soils was investigated by quantifying total fungal ergosterol. Whatever the soil analyzed, no significant differences in total soil fungal biomass between the bioaugmented and the control mesocosms were found after three months of treatment (Figure 3).

### 3.3. Soil Hydrolase Activities

**Figure 4:**
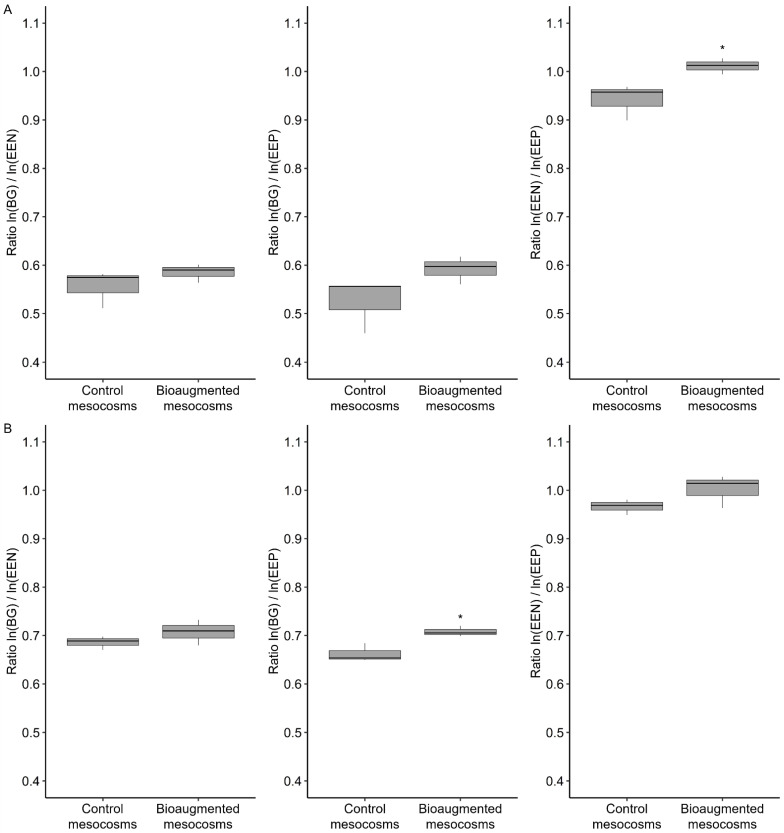
Enzyme logarithmic ratios in the soil H (**A**) and in the soil L (**B**) after a three-month-treatment. The corresponding enzymes are: BG (β-1,4-glucosidase); EEN = NAG (β-1,4-N-acetylglucosaminidase) + LAP (leucine aminopeptidase); EEP = PHOS (phosphomonoesterase). The line within the box shows the median value, the bar lines above and below the boxes indicate minimum and maximum values (*n* = 3). The asterisk indicates significant differences between treatment (Student’s test; *p*-value < 0.05). EEN: extracellular enzymes of hydrolysis of N substrates; EEP: extracellular enzymes of hydrolysis of P substrates.

Four enzymatic activities involved in the hydrolysis of assimilable nutrients from environmental sources of C, N and P (Table S2) were measured to assess the impact of fungal bioaugmentation on soil functioning [30]. The total enzymatic activities of hydrolysis of C substrates (BG), N substrates (EEN = NAG + LAP) and P substrates (EEP = PHOS) as well as their ratios were measured in the both soils H and L. With regard to the soil L (Figure 4A), the ratios of C:N and of C:P enzymatic activities were lower (<0.6) and there was no difference between the bioaugmented and the control mesocosms (Figure 4A). In contrast, the ratio of N:P enzymatic activities was higher in both bioaugmented and control mesocosms and was significantly greater (closer to 1) in the mesocosms bioaugmented with the 5 fungal strains (Figure 4A). Similar profiles were obtained with the soil L but with only the ratio of C:P activities being significantly greater in the bioaugmented mesocosms compared to the control ones, but nonetheless far from 1 (<0.75) (Figure 4B).

## 4. Discussion

While most studies have explored the ability of ligninolytic Basidiomycete strains in the mycoremediation of PCB-polluted soils [2,4,14,15,31] and have shown in most cases underwhelming results [16], very few studies have investigated the potentiality of Ascomycete strains isolated from PCB-polluted soils to degrade these recalcitrant pollutants. These endogenous strains seemed to possess high potential capacities to degrade PCBs in liquid medium comparable to those of ligninolytic basidiomycete strains [17,21,22,32] and could potentially grow easily in their native soil without being hindered by competition with other native strains. In this study, the abilities of the consortium of five Ascomycetes strains, *P. chrysogenum, P. canescens*, *P. citreosulfuratum*, *A. jensenii*, and *Ac. sclerotigenum* [17] in the mycoremediation of historically PCB-polluted soils and their performance to restore the biological functioning of these soils were investigated. Two soils with different PCB concentrations were used to apprehend the inherent properties of these strains to depollute soils.

A significant PCB depletion of 29% in the soil H was evidenced after three months of incubation leading to a decrease of the PCB concentration from 850 ppm to 604 ppm. This is to our knowledge the highest rate of PCB depletion obtained by fungal treatment on such a highly PCB-polluted soil (Σ7 congeners >800 ppm). This degradation induced the modification of the distribution of PCB congeners with the increase of the lowly chlorinated PCB probably resulting from the mineralization of highly chlorinated PCB congeners as reported elsewhere [33,34,35,36].

The PCB degradation obtained by the five fungal strains in the soil H was not correlated with an increase in the total fungal biomass in the bioaugmented mesocosms at the end of treatment. This contrast with previous studies conducted by Sage et al. (2014) [5] showing, via quantitative PCR targeting each strain, a development of some bioaugmented strains after six months of treatment. The lack of an increase in fungal biomass in the bioaugmented mesocosms may be related to the growth decline of the bioaugmented strains that probably occurred after a phase of fungal development within the early stages of treatment. Interestingly, the straw used for the fungal strains’ growth was completely drained in the bioaugmented mesocosms and no longer visible in comparison to the control mesocosms (data not shown). In this hypothesis, the partial bioremediation observed could be the result of poor fungal development at the last stage of treatment and could be improved by the soil fungal re-inoculation during the treatment or the re-amendment with carbon source such as straw. It is also possible that the lack of the total fungal biomass increase in the bioaugmented mesocosms may be due to a decrease in the growth of certain autochthonous strains in favor of that of bioaugmented strains. The ergosterol assay method targeting total soil fungi cannot allow to confirm or invalidate this hypothesis.

While the same profile concerning fungal biomass was obtained with the soil L, no PCB biodegradation was obtained in the bioaugmented mesocosms. This result could be linked to the composition of the soil PCB congeners which may be dominated by more recalcitrant congeners [37]. It is also possible that a strong selective pressure of high PCBs concentrations could act on fungal degradation mechanisms. In this case, the fungal enzymes involved in the PCB biodegradation could be inducible by high PCB concentrations comparable to what is described in the bacterial system [38]. This hypothesis is supported by a study conducted by Stella et al., (2017) [4] showing that the amounts of PCBs degraded by *P. ostreatus* was greater in the more PCB-polluted soils. Furthermore, this confirms the capacity of fungal strains to tolerate high levels of PCBs as shown elsewhere [21,22] unlike bacteria which are also sensitive to high PCB content resulting in poor remediation of highly polluted soils [39,40,41].

To assess the impact of fungal treatment on the soil functioning, the enzymatic activities involved in the hydrolysis of C, N and P derived nutrients were evaluated. The ratios between these different enzymatic activities provided information on the nutritional and metabolic properties of the soil microbial communities and therefore the quality of soil functioning [42]. Better soil functioning is characterized by a ratio for C:N:P enzymatic activities near 1:1:1 [42]. The biodegradation observed in the soil H did not seem to improve the ratios for C:N and C:P activities. They appeared far from equilibrium (much less than 1) and did not vary between different mesocosms [42]. This could be explained by the fact that the soil always remained contaminated with PCBs despite the partial remediation by the five fungal strains. On the other hand, a better nutritional and metabolic activity of the soil microbial communities in the bioaugmented soil concerning the N:P enzymatic activities were obtained and suggested in this case a better functioning of the bioaugmented soil. Results obtained with the soil L, displaying insensitivity to fungal biodegradation, confirmed the link between biodegradation and improving soil functioning. Indeed, the effect of bioaugmentation, although appearing on the C:N enzymatic activities, seemed very limited (C:N enzymatic activities much less than 1) [42].

## 5. Conclusions

The consortium of five native Ascomycete strains isolated from the soil H demonstrated its capacity to partially remediating it by degrading 27% of the seven indicator PCBs. This led to an important decrease of PCBs from 850 ppm to 604 ppm. This remarkable biodegradation capacity was not observed in the soil L, soil from which the strains did not originate. Although an increase in fungal biomass was not evidenced in both soils at the end of the treatment, the biodegradation in the soil H resulted in a significantly improved stoichiometry of N and P acquiring enzymatic activities suggesting the partial restoration of the soil functioning. It would be possible to improve these strains remediation capacities by re-inoculating them into the soil at stake throughout the treatment or re-amending the soil with carbon source (straw) to stimulate the development of fungi. This could contribute to an important step in the mycoremediation of sites polluted by such recalcitrant molecules such as PCBs.

## Figures and Tables

**Table 1:** Physico-chemical characteristics of PCB-polluted soils.

Soil	Σ7 Indicator PCBs (mg kg^−1^)	pH	Total Organic Matter (%)	Composition (%)		
				Sand	Silt	Clay
H	850 ± 89.00	8.21 ± 0.06	3.61 ± 0.13	20	50	30
L	36 ± 1.80	7.80 ± 0.50	2.55 ± 0.11	45	35	20

**Table 2:** Comparison of PCB congener concentrations in bioaugmented and control mesocosms and percentage of degradation of each congener. Asterisk indicates significant differences between treatment (Welch test; *p*-value < 0.05).

PCB Congeners	Control Mesocoms PCBs Residuals (mg kg^−1^)	Bioaugmented Mesocoms PCBs Residuals (mg kg^−1^)	*p*-Value	PCB Depletion (%)
28	0.37 ± 0.01	0.39 ± 0.08	0.8549	−5.41
52	18.53 ± 0.50	24.40 ± 5.81	0.4966	−31.65
101	120.00 ± 4.19	72.17 ± 7.87	0.02128 *	39.86
118	52.63 ± 2.23	46.97 ± 3.74	0.3601	10.77
138	234.67 ± 8.85	169.33 ± 22.16	0.1242	27.84
153	235.67 ± 8.28	172.33 ± 21.94	0.13	26.87
180	171.00 ± 7.13	118.63 ± 16.06	0.1005	30.62

**Table 3:** Comparison of PCB congener concentrations in bioaugmented and control mesocosms and percentage of degradation of each congener.

PCB Congeners	Control Mesocoms PCBs Residuals (mg kg^−1^)	Bioaugmented Mesocoms PCBs Residuals (mg kg^−1^)	*p*-Value	PCB Depletion (%)
28	0.05 ± 0.01	0.06 ± 0.01	0.3868	−12.5
52	0.24 ± 0.04	0.20 ± 0.03	0.2406	17.61
101	0.960 ± 0.24	0.963 ± 0.11	0.5827	−0.26
118	0.410 ± 0.06	0.408 ± 0.04	0.07827	0.61
138	4.99 ± 0.40	5.47 ± 0.38	0.9593	−9.74
153	4.31 ± 0.77	4.85 ± 0.55	0.828	−12.5
180	4.56 ± 0.41	5.63 ± 0.68	0.4705	−23.5

## Supplementary Materials

The following are available online at https://www.mdpi.com/2076-2607/9/3/612/s1, Table S1: Differences between soil types and between PCB congeners; Table S2: Enzymatic activities in nmol g-1 soil h-1 in bioaugmented mesocosms (A) and in control mesocosms (B) after three months of treatment.

## References

[1] Chun S.C., Muthu M., Hasan N., Tasneem S., Gopal J. (2019). Mycoremediation of PCBs by Pleurotus Ostreatus: Possibilities and Prospects. Appl. Sci..

[2] Sharma J.K., Gautam R.K., Nanekar S.V., Weber R., Singh B.K., Singh S.K., Juwarkar A.A. (2018). Advances and Perspective in Bioremediation of Polychlorinated Biphenyl-Contaminated Soils. Environ. Sci. Pollut. Res..

[3] Li Y.-F., Harner T., Liu L., Zhang Z., Ren N.-Q., Jia H., Ma J., Sverko E. (2010). Polychlorinated Biphenyls in Global Air and Surface Soil: Distributions, Air−Soil Exchange, and Fractionation Effect. Environ. Sci. Technol..

[4] Stella T., Covino S., Čvančarová M., Filipová A., Petruccioli M., D’Annibale A., Cajthaml T. (2017). Bioremediation of Long-Term PCB-Contaminated Soil by White-Rot Fungi. J. Hazard. Mater..

[5] Sage L., Périgon S., Faure M., Gaignaire C., Abdelghafour M., Mehu J., Geremia R.A., Mouhamadou B. (2014). Autochthonous Ascomycetes in Depollution of Polychlorinated Biphenyls Contaminated Soil and Sediment. Chemosphere.

[6] Mourier B., Desmet M., Van Metre P.C., Mahler B.J., Perrodin Y., Roux G., Bedell J.-P., Lefèvre I., Babut M. (2014). Historical Records, Sources, and Spatial Trends of PCBs along the Rhône River (France). Sci. Total Environ..

[7] Yadav I.C., Devi N.L., Li J., Zhang G. (2017). Polychlorinated Biphenyls in Nepalese Surface Soils: Spatial Distribution, Air-Soil Exchange, and Soil-Air Partitioning. Ecotoxicol. Environ. Saf..

[8] Géorisques https://www.georisques.gouv.fr/risques/sites-et-sols-pollues/donnees#/type=instructions.

[9] Faroon O., Jones D., De Rosa C. (2000). Effects of Polychlorinated Biphenyls on the Nervous System. Toxicol. Ind. Health.

[10] Bedard D.L. (2008). A Case Study for Microbial Biodegradation: Anaerobic Bacterial Reductive Dechlorination of Polychlorinated Biphenyls—From Sediment to Defined Medium. Annu. Rev. Microbiol..

[11] Lauby-Secretan B., Loomis D., Grosse Y., Ghissassi F.E., Bouvard V., Benbrahim-Tallaa L., Guha N., Baan R., Mattock H., Straif K. (2013). Carcinogenicity of Polychlorinated Biphenyls and Polybrominated Biphenyls. Lancet Oncol..

[12] Stølevik S.B., Nygaard U.C., Namork E., Haugen M., Meltzer H.M., Alexander J., Knutsen H.K., Aaberge I., Vainio K., van Loveren H. (2013). Prenatal Exposure to Polychlorinated Biphenyls and Dioxins from the Maternal Diet May Be Associated with Immunosuppressive Effects That Persist into Early Childhood. Food Chem. Toxicol..

[13] Anitescu G., Tavlarides L.L. (2006). Supercritical extraction of contaminants from soils and sediments. J. Supercrit. Fluids.

[14] Sadañoski M.A., Benítez S.F., Fonseca M.I., Velázquez J.E., Zapata P.D., Levin L.N., Villalba L.L. (2019). Mycoremediation of High Concentrations of Polychlorinated Biphenyls with Pleurotus Sajor-Caju LBM 105 as an Effective and Cheap Treatment. J. Environ. Chem. Eng..

[15] Sadañoski M.A., Benitez S.F., Velázquez J.E., Fonseca M.I., Zapata P.D., Levin L.N., Villalba L.L. (2020). Bioprocess Conditions for Treating Mineral Transformer Oils Contaminated with Polychlorinated Biphenyls (PCBs). J. Environ. Chem. Eng..

[16] Harms H., Schlosser D., Wick L.Y. (2011). Untapped Potential: Exploiting Fungi in Bioremediation of Hazardous Chemicals. Nat. Rev. Microbiol..

[17] Germain J., Raveton M., Binet M.N., Mouhamadou B. (2021). Screening and Metabolic Potential of Fungal Strains Isolated from Contaminated Soil and Sediment in the Polychlorinated Biphenyl Degradation. Ecotoxicol. Environ. Saf..

[18] Cruz-Izquierdo R.I., Paz-González A.D., Reyes-Espinosa F., Vazquez-Jimenez L.K., Salinas-Sandoval M., González-Domínguez M.I., Rivera G. (2021). Analysis of Phenanthrene Degradation by Ascomycota Fungi Isolated from Contaminated Soil from Reynosa, Mexico. Lett. Appl. Microbiol..

[19] Siracusa G., Yuan Q., Chicca I., Bardi A., Spennati F., Becarelli S., Levin D.B., Munz G., Petroni G., Di Gregorio S. (2020). Mycoremediation of Old and Intermediate Landfill Leachates with an Ascomycete Fungal Isolate, Lambertella Sp. Water.

[20] González-Abradelo D., Pérez-Llano Y., Peidro-Guzmán H., Sánchez-Carbente M.d.R., Folch-Mallol J.L., Aranda E., Vaidyanathan V.K., Cabana H., Gunde-Cimerman N., Batista-García R.A. (2019). First Demonstration That Ascomycetous Halophilic Fungi (Aspergillus Sydowii and Aspergillus Destruens) Are Useful in Xenobiotic Mycoremediation under High Salinity Conditions. Bioresour. Technol..

[21] Mouhamadou B., Faure M., Sage L., Marçais J., Souard F., Geremia R.A. (2013). Potential of Autochthonous Fungal Strains Isolated from Contaminated Soils for Degradation of Polychlorinated Biphenyls. Fungal Biol..

[22] Périgon S., Massier M., Germain J., Binet M.-N., Legay N., Mouhamadou B. (2019). Metabolic Adaptation of Fungal Strains in Response to Contamination by Polychlorinated Biphenyls. Environ. Sci. Pollut. Res..

[23] Galzy P., Slominski P. (1957). Variations Physiologiques de La Levure Au Cours de La Croissance Sur l’acide Lactique Comme Seule Sorce de Carbone. Comptes Rendus Académie Sci..

[24] Petrić I., Hršak D., Fingler S., Udiković-Kolić N., Bru D., Martin-Laurent F. (2011). Insight in the PCB-Degrading Functional Community in Long-Term Contaminated Soil under Bioremediation. J. Soils Sediments.

[25] Viisimaa M., Karpenko O., Novikov V., Trapido M., Goi A. (2013). Influence of Biosurfactant on Combined Chemical–Biological Treatment of PCB-Contaminated Soil. Chem. Eng. J..

[26] AFNOR Groupe https://www.boutique.afnor.org/norme/nf-en-16167/sols-biodechets-traites-et-boues-dosage-des-polychlorobiphenyles-pcbs-par-chromatographie-en-phase-gazeuse-spectrometrie-gazeuse/article/906803/fa190960.

[27] Gong P., Guan X., Witter E. (2001). A Rapid Method to Extract Ergosterol from Soil by Physical Disruption. Appl. Soil Ecol..

[28] Piton G., Foulquier A., Martínez-García L.B., Legay N., Hedlund K., Martins da Silva P., Nascimento E., Reis F., Sousa J.P., De Deyn G.B. (2020). Disentangling Drivers of Soil Microbial Potential Enzyme Activity across Rain Regimes: An Approach Based on the Functional Trait Framework. Soil Biol. Biochem..

[29] The R Project for Statistical Computing. https://pbil.univ-lyon1.fr/CRAN/.

[30] Lee S.-H., Kim M.-S., Kim J.-G., Kim S.-O. (2020). Use of Soil Enzymes as Indicators for Contaminated Soil Monitoring and Sustainable Management. Sustainability.

[31] Federici E., Giubilei M., Santi G., Zanaroli G., Negroni A., Fava F., Petruccioli M., D’Annibale A. (2012). Bioaugmentation of a Historically Contaminated Soil by Polychlorinated Biphenyls with Lentinus Tigrinus. Microb. Cell Factories.

[32] Tigini V., Prigione V., Di Toro S., Fava F., Varese G.C. (2009). Isolation and Characterisation of Polychlorinated Biphenyl (PCB) Degrading Fungi from a Historically Contaminated Soil. Microb. Cell Factories.

[33] Vyas B.R.M., Šašek V., Matucha M., Bubner M. (1994). Degradation of 3,3′,4,4′-Tetrachlorobiphenyl by Selected White Rot Fungi. Chemosphere.

[34] Dietrich D., Hickey W.J., Lamar R. (1995). Degradation of 4,4′-Dichlorobiphenyl, 3,3′,4,4′-Tetrachlorobiphenyl, and 2,2′,4,4′,5,5′-Hexachlorobiphenyl by the White Rot Fungus Phanerochaete Chrysosporium. Appl. Environ. Microbiol..

[35] Beaudette L.A., Davies S., Fedorak P.M., Ward O.P., Pickard M.A. (1998). Comparison of Gas Chromatography and Mineralization Experiments for Measuring Loss of Selected Polychlorinated Biphenyl Congeners in Cultures of White Rot Fungi. Appl. Environ. Microbiol..

[36] Beaudette L.A., Ward O.P., Pickard M.A., Fedorak P.M. (2000). Low Surfactant Concentration Increases Fungal Mineralization of a Polychlorinated Biphenyl Congener but Has No Effect on Overall Metabolism. Lett. Appl. Microbiol..

[37] Stella T., Covino S., Burianová E., Filipová A., Křesinová Z., Voříšková J., Větrovský T., Baldrian P., Cajthaml T. (2015). Chemical and Microbiological Characterization of an Aged PCB-Contaminated Soil. Sci. Total Environ..

[38] Passatore L., Rossetti S., Juwarkar A.A., Massacci A. (2014). Phytoremediation and Bioremediation of Polychlorinated Biphenyls (PCBs): State of Knowledge and Research Perspectives. J. Hazard. Mater..

[39] Borja J., Taleon D.M., Auresenia J., Gallardo S. (2005). Polychlorinated Biphenyls and Their Biodegradation. Process Biochem..

[40] Vasilyeva G.K., Strijakova E.R. (2007). Bioremediation of Soils and Sediments Contaminated by Polychlorinated Biphenyls. Microbiology.

[41] Yang Y., Chen S., Li S., Chen M., Chen H., Liu B. (2014). Bioleaching Waste Printed Circuit Boards by Acidithiobacillus Ferrooxidans and Its Kinetics Aspect. J. Biotechnol..

[42] Sinsabaugh R.L., Hill B.H., Follstad Shah J.J. (2009). Ecoenzymatic Stoichiometry of Microbial Organic Nutrient Acquisition in Soil and Sediment. Nature.

