# Supplementary material for: Potentiality of Native Ascomycete Strains in Bioremediation of Highly Polychlorinated Biphenyl Contaminated Soils
Source: Microorganisms. 2021 Mar 16;9(3):612. doi: 10.3390/microorganisms9030612 (PMC8002370; doi:10.3390/microorganisms9030612)
Supplement: Supplementary file 1 [file microorganisms-09-00612-s001.pdf]

Supplementary materials

SM1: Differences between soil types and between PCB congeners

Variables	Df	Sum sq	Mean sq	F value	Pr(>F)
Soil type	1	11203	11203	22.332	5.87×10^{-5} ***
PCB congeners	6	250838	41806	83.338	2×10^{-16} ***
Soil type x PCB congeners	6	8862	1477	2.944	0.0235 *
Residuals	28	14046	502		

SM2. Enzymatic activities in nmol g^{-1} soil h^{-1} in bioaugmented mesocosms (A) and in control mesocosms (B) after three months of treatment. The corresponding enzymes are BG : β-1,4-glucosidase, LAP : L-leucine aminopeptidase, NAG : β-1,4-N-acetylglucosaminidase, PHOS : Phosphatases, EEN : sum of NAG and LAP. EEN: extracellular enzymes of hydrolysis of N substrates; EEP: extracellular enzymes of hydrolysis of P substrates. Values are not significantly different (Welch test; p-value >0.05).

A

Enzymes	Control mesocosms	Bioaugmented mesocosms	p-value
BG	42.97 ± 7.16	61.37 ± 7.07	0.2097
LAP	773.86 ± 89.06	1082.37 ± 108.56	0.15
NAG	28.22 ± 8.67	30.01 ± 2.39	0.8838
EEP = PHOS	1183.42 ± 26.29	1016.49 ± 49.13	0.09039
EEN = LAP + NAG	802.08 ± 97.03	1112.38 ± 110.88	0.1619

B

Enzymes	Control mesocosms	Bioaugmented mesocosms	p-value
BG	54.50 ± 4.93	58.25 ± 1.06	0.6013
LAP	315.22 ± 33.59	294.94 ± 42.52	0.7759
NAG	25.28 ± 1.52	27.98 ± 1.01	0.3029
EEP = PHOS	412.32 ± 25.74	313.54 ± 19.81	0.07208
EEN = LAP + NAG	340.50 ± 34.85	322.91 ± 41.64	0.8049
